# A Treatise on Fractures

**Published:** 1859-01

**Authors:** 


ART. IV.—*A Treatise on Fractures.* By J. F. MALGAIGNE, Chirurgien de l'Hôpital Saint-Louis, Chevalier de la Legion d'Honneur et du Mérite Militaire de Pologne, Membre de l'Académie Royale de Médecine. With one hundred and six illustrations. Translated from the French, with notes and additions, by JOHN H. PACKARD, M.D. Philadelphia: J. B. Lippincott & Co., 1859. Octavo, pp. 684.

THE Treatise on Fractures of M. Malgaigne, now Officer of the Legion of Honor and Professor of Operative Surgery in the Paris School of Medicine, was published in 1847. The author states in his preface that it was written with the object of filling a gap in French surgical literature. He accomplished his intended purpose, and more than did so, for the work he has erected must be regarded as a monument, conspicuous and to be admired, even among the noble monuments of the medical literature of his country. As a solid, complete, substantial, highly finished work, we know of none that is its superior; it can, with justice, be regarded as a model in scientific literature.

It is a work, also, so well known to many of the readers of this REVIEW that we almost hesitate to present in its pages any analysis of its contents; and feel inclined to do no more than announce an American translation, and add some observations on the notes and additions of the translator, and the manner in which he has performed his task. As, however, there are many readers who, until the publication of this translation, have been debarred from its acquaintance, by want of familiarity with the language in which it was written, we will make a brief statement of its contents.

The whole volume is divided into nineteen chapters; one of them, the first, treats of fractures in general; the others, of fractures of particular bones.

The first chapter treating, as just said, of fractures in general, occupies 271 out of the whole 660 pages occupied by the text of the book. It is divided into seven separate articles, devoted respectively to the causes of fractures, their varieties, their general symptoms, their course and terminations, diagnosis, prognosis, and treatment. Each article is divided again into a number of separate paragraphs; for example, article iv., or that treating of the course and terminations of fractures, is divided into five paragraphs, treating respectively of the exterior phenomena, of the formation of the callus, of the transformations of the callus, the theory of anchylosis following fracture, and of non-union or false-joint after fracture. The chapters devoted to the fractures of particular bones are equally complete and arranged with equal care and method. That upon fractures of the femur, for example, is divided into as many as nine separate paragraphs, treating respectively of intra-capsular fractures of the neck, extra-capsular fractures of the neck, fractures of the great trochanter, fractures just below the trochanters, fractures in the middle third of the femur, fractures just above the condyles, fractures of one condyle, fractures of both condyles at once, and compound fractures of the femur.

The great character which M. Malgaigne sought to give his work was that of *reality;* that is, he desired to make it a work of truth—not of the imagination, a work founded upon facts—not one composed merely of theories. "In a historical point of view," he says, "I have aimed at presenting a *resumê* of all the doctrines and ideas maintained from the earliest times to our own day, having recourse as much as possible to the original works. As to my teachings, I have asserted nothing unsupported by facts, drawn either from my own experience or from that of others. When clinical observation was insufficient, I have sought a substitute for it in experiments upon dead bodies, or upon the lower animals; but, above all, I have tried to clear up a great many mooted points by throwing upon them the light of pathological anatomy; and this is the object of my atlas," (p. 16.) For the task he has accomplished so well, M. Malgaigne was, in many respects, peculiarly fitted. He is a man not only of thorough study, of extensive learning, and of prodigious industry, but he is also a man who thinks for himself; he is noted in France as a man of

*opinions tranchées*, a man decided in his opinions. It is always satisfactory in a dogmatic treatise, whatever may be the array of authorities cited, to have also given that of the author himself. Among the authorities repeatedly mentioned in this work, it is a matter of real gratification to remark the frequency with which the names of some of our own most distinguished surgeons occur, particularly those of Physick and Norris, and the consideration accorded to their opinions and statements.

In presenting to the profession his translation of the work of Malgaigne, Dr. Packard says:—

"I have made it my great aim to render the text of the author as faithfully as possible, endeavoring at the same time to avoid offending the taste of the reader by the use of Gallic idioms. The notes which I have taken the liberty to insert are intended to set forth peculiarities in American views and practice, or accounts of cases in point; in one or two instances I have been able to look up quotations which were beyond my author's reach. An index has also been added, and a list, as full as circumstances would allow, of works hitherto published on the same subject."

We look upon Dr. Packard's translation of the French text as a very excellent one; we have examined it with care, and find that the original text has always been correctly and faithfully rendered. The faults we could detect were very trifling; the French *grand* is sometimes translated as *grand*, when *great* would be more proper, as at page 617, where we read that "all apparatus of this kind have two grand defects." We doubt, also, whether it is correct to use *reserved* as the French use réservé. We may say that "it is proper to be reserved in the prognosis," but we question whether it can be said correctly that "the prognosis should therefore be to a certain point reserved," (p. 627.)

The notes which have been inserted for the purpose of setting forth peculiarities in American views and practice are valuable, and fulfill completely the object for which they were introduced. The accounts of cases that have been inserted are interesting and instructive; and the cases themselves are always to the point, at the time in question. These cases have generally fallen under Dr. Packard's own observation, in the Pennsylvania Hospital.

In a few instances Dr. Packard has introduced notes, with an object not mentioned in the preface, namely, the statement of an opinion of his own, as somewhat different from that recorded by M. Malgaigne. At page 210, the note introduced reads as follows:—

"The operation of bending the callus of a broken bone is one by no means free from pain, or even danger; and the longer the adjacent joints are kept at rest, the greater the pain and difficulty of making passive motion in them. Moreover, not only is the approach of gangrene oftentimes extremely insidious, but the repair of a fracture is materially favored by a sound and transpirable condition of the skin overlying it. From these considerations it may be seen that the advantages attending the frequent examination and re-dressing of broken limbs are very great; so great as in the opinion of most surgeons to counterbalance the risk of disturbing the position of the fragments, which, after all, may be obviated by using due caution. *Constant and careful watching of the fracture from the outset*, has saved many a patient from permanent crippling."

This note was called for, and we think that the opinion of Dr. Packard in regard to frequent dressings in cases of fracture will be readily accepted as the correct one. It is, however, the only one of the notes in question, of which we are able to say this; the others appear to us to be unnecessary and the opinions they express of doubtful accuracy. They are as follows:—

Speaking of the effect of pressure, in fractures of the lower extremity, the patient being confined to bed, M. Malgaigne says:—

"In very fleshy subjects, the danger is augmented by their weight, but it is also more easily counteracted; for we need only have the board between the mattrasses, as proposed by J. L. Petit, lengthened so as to reach a little beyond the buttock, and widened so as to equal the bed, in order to give an equable support to the pelvis and limbs. If, on the contrary, the patient is spare, giving reason to fear the effect of pressure upon the sacrum by the board, we must be satisfied with supporting the sound side by means of cushions, after Duverney's method, but with the indispensable precaution of daily watching and arranging them," (p. 156.)

Dr. Packard then introduces a note to say:—

"It may be doubted whether the mere weight of the patient does not tell equally upon the skin where there is a thick layer of fat *above* it, and where there is but little; and perhaps the advantage of that layer, however great, may be counterbalanced by the additional weight which its pressure involves."

After what Malgaigne had said, this note was not needed, and few persons will doubt with Dr. Packard as to the effect of compressing the skin between a bone and a board, and a board and a mass of fat. All his own doubts would be at once dissipated by simply changing the resting spot of his body from his buttocks to his shins. The skin covering the latter, be it remembered, is endowed with no more acute sensibility than that over the former.

At page 666, the text says:—"M. Rognetta says that he twice saw simple fractures of the astragalus, without any displacement or any injury of the soft parts; at the instant of the accident, or even after the swelling had subsided, he detected the lesion by the sensation imparted to the touch, *like that of several nuts in a bag*."

The notes add:—"It may be doubtful whether the multiple character of these fractures was proved by the sensation alluded to, since it is well known how completely the most delicate touch may be deceived; and especially where effusion into the articular cavity would increase the error." It is stated expressly that the sensation was felt at the instant of the accident, or even after the swelling had subsided, or at a time when effusion into the articular cavity was not increasing the chances of error dependent upon mistakes to be made by the touch, so well known that any reference to them in this general way was unnecessary.

At page 351, M. Malgaigne relates a curious case from Sabatier, where "the last two true ribs had been fractured by the passage of a carriage-wheel; but the lower one was broken in two places, and in such a way that the middle fragment, being quite movable, was drawn into the chest in inspiration, and pushed outward in expiration." "Probably," says the note, "the author meant just the reverse of this; the fragment would be *pushed outward in inspiration, drawn in, in expiration.*" A difference of opinion can only exist here from contrary views as to the manner in which respiration is performed. M. Malgaigne reasons as if the air entered the chest to fill the vacuum made by the movement of the walls in inspiration, and we shall prefer his view of the matter.

At page 37 the text reads thus:—"A young plasterer, driving his cart along a very muddy road, had put his foot in a deep rut; at the same moment he tried to whip up his horses; the blow missed; he was dragged forward, nearly fell, and had his leg broken by the edge of the rut." The note says:—"Might not two forces be said to have acted in this instance on the ends and middle of the bone? The weight of the body would act above; the bottom of the rut would fix the foot, and the edge of the rut bear against the middle of the leg; the latter would be in fact the agent producing the fracture."

The case was cited by M. Malgaigne in order to exemplify the effects of flexion, applied to the continuity of a curved bone, just as when an attempt is made to bend a stick over the knee. In the flexion in question, he adds, "the bone is held fast particularly by one certain point of its continuity, and the force is exercised upon one of its extremities or upon both at once." Any note for the purpose of explaining the manner in which the fracture occurred, was, under the circumstances, unnecessary.

At page 81 of the French edition, M. Malgaigne, after speaking of the *complications of fracture*, ends the paragraph thus:—"Et enfin les chirurgiens Anglais ont fini par restraindre la denomination de *fractures compliqueés* à celles seulement qui, par une plaie des téguments, sont exposeés au contact de l'air." In English we should make this to say that "the English surgeons call only those fractures *compound* that communicate by a wound with the external air." At page 79 of the translation,

Dr. Packard, supposing that here *compliqueés* should be translated not as *compound*, but as *complicated*, adds a note expressive of surprise that M. Malgaigne could fall into an error, into which, we think, he has not fallen, in regard to the meaning attached by English writers to the words *compound* and *complicated*.

We have now noticed all the faults we could find in this American edition of Malgaigne, and we have searched diligently for them. They are certainly not very grievous, and it may be thought that in mentioning what we have we manifest a fault-finding spirit. We have always found M. Malgaigne's opinions so correct, and his mode of expressing them so clear, that we confess to feeling annoyed at attempts to differ from him, or to render his meaning more evident.

It remains for us to say that the bibliography added by Dr. Packard, and which M. Malgaigne had omitted, notwithstanding the statement made in his preface, is quite extensive. The plates "which, for facility of reference, have been collected at the end of the volume, instead of being interspersed throughout the text," we have compared with those in M. Malgaigne's truly admirable folio atlas. They are reduced in size one-half, and are wood engravings, while the original are lithographs, and of exquisite execution. Still they must be considered as good illustrations, and they certainly are accurate copies of the original plates.

The handsome manner in which the work has been published deserves special acknowledgment.

